# Exploratory Hydrocarbon Drilling Impacts to Arctic Lake Ecosystems

**DOI:** 10.1371/journal.pone.0078875

**Published:** 2013-11-06

**Authors:** Joshua R. Thienpont, Steven V. Kokelj, Jennifer B. Korosi, Elisa S. Cheng, Cyndy Desjardins, Linda E. Kimpe, Jules M. Blais, Michael FJ. Pisaric, John P. Smol

**Affiliations:** 1 Paleoecological Environmental Assessment and Research Lab (PEARL), Department of Biology, Queen's University, Kingston, Ontario, Canada; 2 Northwest Territories Geoscience Office, Government of the Northwest Territories, Yellowknife, Northwest Territories, Canada; 3 Department of Biology, University of Ottawa, Ottawa, Ontario, Canada; 4 Department of Geography and Environmental Studies, Carleton University, Ottawa, Ontario, Canada; 5 Department of Geography, Brock University, St. Catharines, Ontario, Canada; University of Kansas, United States of America

## Abstract

Recent attention regarding the impacts of oil and gas development and exploitation has focused on the unintentional release of hydrocarbons into the environment, whilst the potential negative effects of other possible avenues of environmental contamination are less well documented. In the hydrocarbon-rich and ecologically sensitive Mackenzie Delta region (NT, Canada), saline wastes associated with hydrocarbon exploration have typically been disposed of in drilling sumps (i.e., large pits excavated into the permafrost) that were believed to be a permanent containment solution. However, failure of permafrost as a waste containment medium may cause impacts to lakes in this sensitive environment. Here, we examine the effects of degrading drilling sumps on water quality by combining paleolimnological approaches with the analysis of an extensive present-day water chemistry dataset. This dataset includes lakes believed to have been impacted by saline drilling fluids leaching from drilling sumps, lakes with no visible disturbances, and lakes impacted by significant, naturally occurring permafrost thaw in the form of retrogressive thaw slumps. We show that lakes impacted by compromised drilling sumps have significantly elevated lakewater conductivity levels compared to control sites. Chloride levels are particularly elevated in sump-impacted lakes relative to all other lakes included in the survey. Paleolimnological analyses showed that invertebrate assemblages appear to have responded to the leaching of drilling wastes by a discernible increase in a taxon known to be tolerant of elevated conductivity coincident with the timing of sump construction. This suggests construction and abandonment techniques at, or soon after, sump establishment may result in impacts to downstream aquatic ecosystems. With hydrocarbon development in the north predicted to expand in the coming decades, the use of sumps must be examined in light of the threat of accelerated permafrost thaw, and the potential for these industrial wastes to impact sensitive Arctic ecosystems.

## Introduction

The Mackenzie Delta in Canada's western Arctic is underlain by significant discovered and predicted reserves of hydrocarbons [1], but is also amongst the most rapidly warming regions globally [2]. Activities associated with the exploitation of these hydrocarbon resources, including enhanced exploration, as well as infrastructure development through the extraction, production and transmission of hydrocarbons to market, constitute an additional stressor to the freshwater ecosystems of the region. The Mackenzie Delta region is ecologically important, as identified by the establishment of the Kendall Island Migratory Bird Sanctuary in 1961, as well as being culturally significant for local indigenous communities [3]. Much recent attention has focused on oil and gas activities increasing the delivery of toxic polycyclic aromatic hydrocarbons (PAHs) into the environment [4], [5]; however the potential effects of industrial activities on aquatic ecosystems are widespread, and PAH contamination is just one example of the environmental consequences of oil and gas exploration and development.

**Figure 1:**
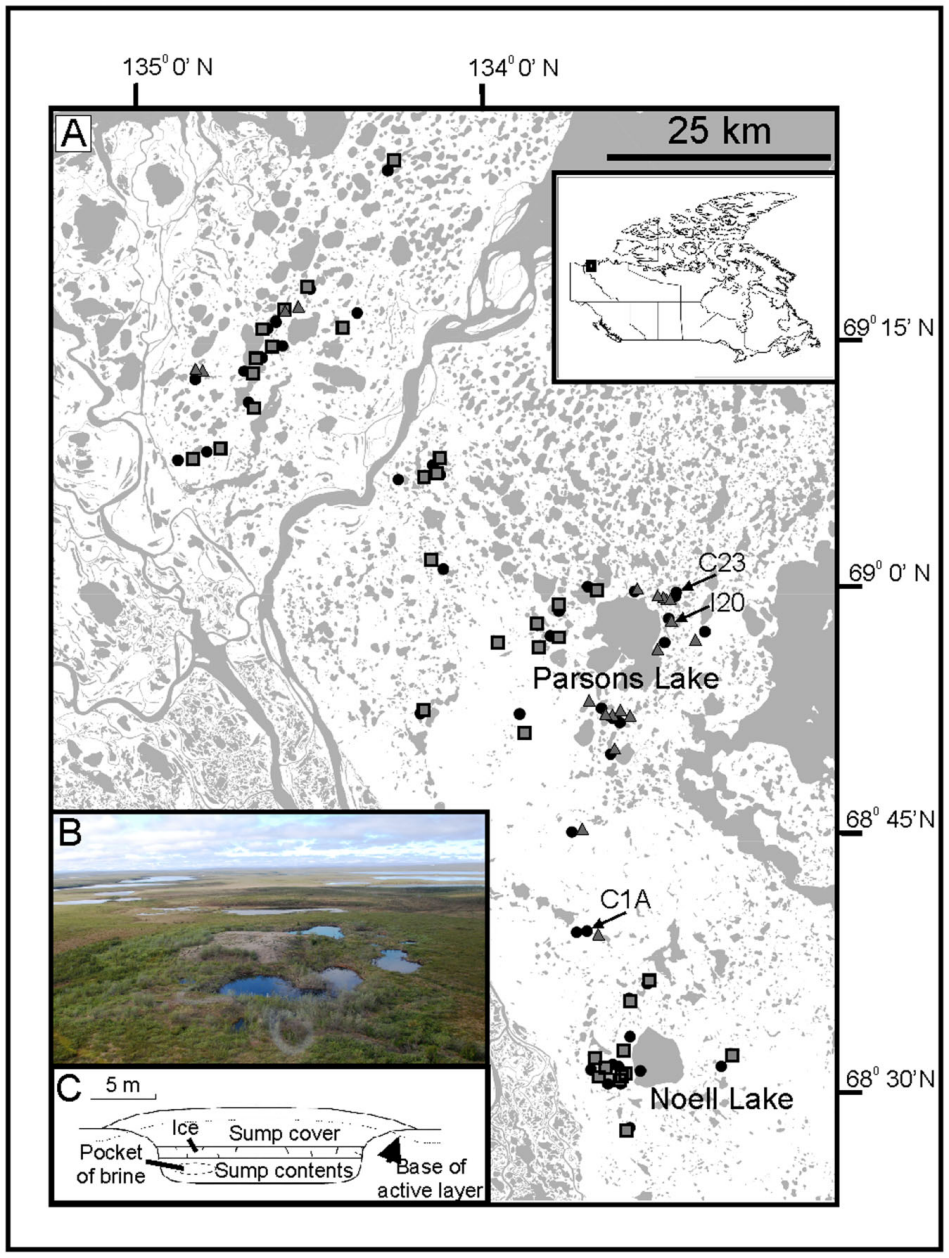
Map of study area, schematic of typical drilling sump and image of a degrading sump. A) The location of the 101 study lakes in the Mackenzie Delta uplands (Northwest Territories, Canada) (triangles – drilling-sump lakes; squares – thaw-slump lakes; circles – control lakes). Inset shows the region in the context of Canada. B) Image of a degrading drilling sump from the Mackenzie Delta uplands, near Parsons Lake, exhibiting significant surface and perimeter ponding. C) Generalized schematic of a drilling mud sump. A large pit is excavated into the permafrost and filled with the drilling wastes and fluids. These drilling fluids are then allowed to partially or completely freeze, and backfilled with the excavated material. The assumption is that the material will be permanently contained in the permafrost. Redrawn from [8].

Hydrocarbon exploration has been occurring in the Mackenzie Delta region (Fig. 1a) since the 1960s, and was particularly intense during the 1970–80s and around 2000 [6]. In the Canadian Arctic, wastes associated with the drilling of exploratory onshore hydrocarbon wells have been typically disposed of using in-ground sumps (Fig. 1b, c; [7]). These large excavations into the permafrost, usually located next to the exploratory well, are intended to act as a permanent containment location for the wastes produced during exploratory well development, including mud and rock cuttings, and drilling fluids. Drilling fluids are made up of, surfactants and detergents, as well as large quantities of highly concentrated saline solutions (primarily potassium chloride, with concentrations up to 100 g L^-1^), which are used as a freezing point depressant during winter drilling operations [8]–[10]. Historically, a typical three-kilometre deep well, required approximately 40,000 m^3^ of drilling fluid alone [7], though this volume has been reduced in more recent operations due to an improvement in recycling technologies.

More than 150 drilling sumps have been constructed in the Mackenzie Delta region since the mid-1960s [6]. As most exploration activity occurs in the winter (this practice has been required by law since 1986 in an attempt to minimize the environmental impact of drilling activities), these fluids are meant to freeze *in situ*, and then capped with the material excavated from the sump. It was assumed the drilling muds are permanently encapsulated within the surrounding permafrost, and that drilling sumps represent a permanent containment mechanism for materials associated with hydrocarbon exploration [7].

Pronounced climate warming in the western Canadian Arctic has serious implications for sump containment, as increasing air temperatures are increasing tall shrub cover and causing permafrost temperatures to rise [3]. Recent studies of sump integrity have observed increased conductivity beyond the extent of the sump at 74% of the sites studied [6], and as many as one third of these sites are exhibiting surface ponding, suggesting significant thaw of the sump contents has occurred [9]. Vegetation communities growing on sump caps were found to be distinct from surrounding, undisturbed areas, related to factors including drainage, active-layer depth and soil salt concentrations [10]. Increased shrub growth on sump covers enhances snow accumulation and accelerates warming of the sump and lease areas [11]. No assessment of the impacts of saline drilling wastes on nearby freshwater ecosystems exists, although the Mackenzie Delta region and Tuktoyaktuk Coastlands are amongst the most lake-rich environments in the Arctic. This is particularly important, given that many of the compounds present in drilling muds (e.g. KCl, caustic soda, barite) are known to be toxic to freshwater organisms at the concentrations common in drilling muds [7], [12], [13], and because the majority of the drilling sumps constructed are in the catchments of lakes in this water-rich landscape. Understanding the effects of drilling sump failure is essential to evaluate the relative impact of environmental stressors on freshwater in this region.

In this study, we use a combination of contemporary limnological sampling and inferences of past conditions using material preserved in lake sediments (i.e. paleolimnology) to assess the impacts of drilling fluids on the freshwater ecosystems of the Mackenzie Delta uplands region. We compare contemporary water chemistry measurements of lakes with drilling sumps in their catchments to lakes undergoing another major stressor, intense permafrost degradation in the form of retrogressive thaw slumping, as well as undisturbed control lakes with no evidence of localized disturbance. Retrogressive thaw slumps are a common form of thermokarst, which currently occur on the shoreline of approximately 10% of the lakes in the Mackenzie Delta uplands, and are increasing in size and growth rate as a result of recent warming [14]. Thaw slumping is known to result in major changes to contemporary lakewater chemistry [15], [16] and biology [17]. Our previous diatom-based paleolimnological study showed that the main biological response to permafrost thaw in the lakes of the region was related to changes in aquatic habitat availability and water clarity[18]. In this study we expand on our previous sediment-based research in order to understand the impact of hydrocarbon exploration on the lakes of this sensitive region. We used a large present-day limnological dataset of 101 sites to compare lakes with drilling sumps in their catchments to permafrost thaw-affected lakes which are known to be highly-disturbed aquatic systems in the region. Due to the fact that drilling fluids are saline, we hypothesized that if migration of drilling fluids to receiving surface waters has occurred, we should record elevated levels of major ions and conductivity in impacted lakes. Also, due to a lack of long-term biomonitoring data, a variety of sedimentary proxies were analyzed in sediment cores from three lakes in order to put the timing and nature of any limnological change in a historical context. We further hypothesized that if saline-rich wastes from drilling sumps are impacting lakes, shifts in the assemblages of sentinel biological indicators should be detected.

## Materials and Methods

One hundred and one lakes (20 drilling sump, 34 permafrost thaw slump, and 47 control lakes) in the Mackenzie Delta uplands (NT, Canada) were sampled for their present-day limnological conditions in the summers of 2005 or 2007. Thirteen measured physical and chemical variables observed above detection limits in the majority of the sites were selected and normalized. Principal components analysis (PCA) was conducted using the vegan package [19] for R [20]. Analysis of similarity (ANOSIM; [21]) and similarity percentages (SIMPER) were conducted using PRIMER v.6 in order to assess the relationships between the three *a priori* assigned groups, and to determine the variables that contributed to any dissimilarity.

Sediment cores were obtained from lakes I20 (68.9742°N, 133.5253°W; sump-impacted), C23 (68.9978°N, 133.5129°W; control), and C1A (68.6589°N, 133.7602°W; control) (all names unofficial) in August 2009 or July 2010 and sectioned at 0.5 cm resolution using our standard, high-resolution paleolimnological techniques [18]. Lake I20 is located downslope of a drilling sump that shows cover subsidence indicative of permafrost degradation. Lakes C23 and C1A are located nearby in similar terrain to the impacted lake I20, but with no drilling sumps in their respective catchments, and are thus classified as control lakes. Sediment age determination was conducted using ^210^Pb and ^137^Cs radiometric techniques (Fig. S1) [22], following which sedimentary subfossil indicators were isolated and analyzed using standard methods (diatoms: [23]; cladocerans: [24]). Relative percentage diagrams were generated using Tilia v.1.7.16 [25]. Constrained incremental sums of squares (CONISS) cluster analyses were conducted in order to identify biostratigraphic zones of change [26], with the broken stick model used to determine the number of significant zones [27]. Overall lake primary production was estimated by inferring sedimentary chlorophyll *a* concentrations via visual reflectance spectroscopy [28]. PAHs were extracted from wet sediments using accelerated solvent extraction (ASE, Dionex). Approval for all field-based research and the issuing of relevant scientific research licenses was conducted through the Aurora Research Institute (Inuvik, NT). No protected areas or protected species were sampled as part of this research. A more detailed description of the methods used in this study is presented in Text S1.

## Results and Discussion

**Figure 2:**
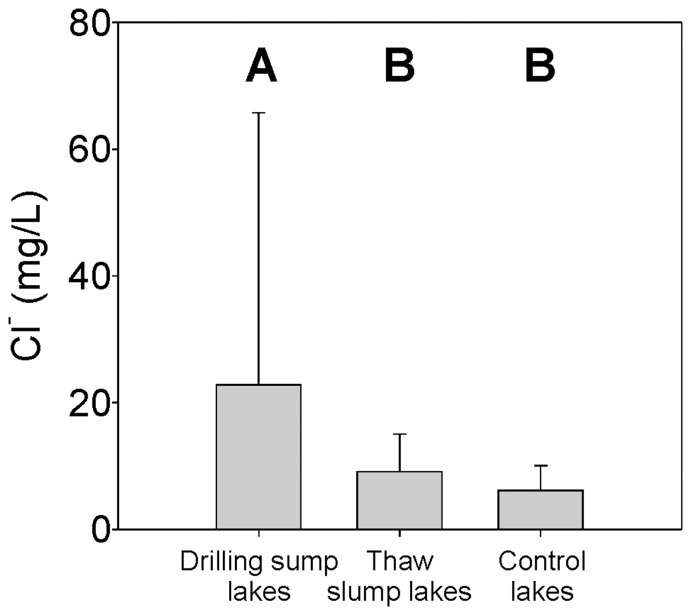
Mean chloride concentration in the 101 lake dataset separated into three *a priori* defined groups. Vertical error bars representing the standard deviation are included. Letters A and B indicate drilling-sump impacted lakes have significantly higher chloride levels than either thaw-slump affected lakes or control lakes, which are not statistically different (Tukey HSD post-hoc test, following ANOVA run on normalized environmental data)

The three *a priori*-defined groups (drilling sump, permafrost thaw slump and control lakes) were found to be significantly different based on their present-day water chemistry (ANOSIM, Global R = 0.307, p = 0.001), and lakes with drilling sumps in their catchments had significantly higher concentrations of chloride (Cl^−^) than either the control lakes or lakes impacted by permafrost thaw slumps (ANOVA, df = 2, 98, F = 7.91, p<0.001; Tukey's HSD post-hoc test) (Fig. 2). The glaciogenic sediments of the Mackenzie Delta uplands are primarily derived from shale bedrock, and as such are naturally high in sulphate, but relatively low in chloride [29], [30]. As a result of permafrost thaw-slump development, the influx of terrestrially-derived materials to lakes results in a significant difference in SO_4_^−2^ concentration, but no significant difference in Cl^−^ concentrations (Fig. S2, Table S1). Significantly greater Cl^−^ concentrations in the drilling-sump impacted lakes (Fig. 2), therefore, suggests a source other than the underlying geological material, such as the wastes from drilling activity, which in most cases are brine-based (e.g. KCl) [7]. Based on similarity percentages (SIMPER), Cl^−^ concentrations contributed 19% of the difference between the drilling-sump and control lakes (more than any other variable), and 13% of the difference between the drilling-sump and thaw-slump lakes (Table S2). Complete comparisons of the measured physical and chemical variables are presented in Text S1. These results indicate that not only is chloride elevated in drilling-sump impacted lakes, but it is also the single most important environmental variable distinguishing sump-impacted lakes from the undisturbed control sites in the region.

Leaching of drilling fluids is clearly the most plausible explanation for the trends in the present-day chemical conditions observed in the drilling-sump impacted lakes in this dataset. Lakes impacted by drilling sumps also exhibited significantly higher concentrations of Ca^2+^, Na^+^, and specific conductivity compared to the control sites, though significantly less than lakes with thaw slumps (Fig. S1). Drilling fluids are the most likely source of these ions to impacted lakes, as materials such as caustic soda (NaOH) are common components [12]. In addition, as drilling fluids migrate through the active layer downslope to lake ecosystems, other ions present in soils would be translocated to the lake. This impact is exacerbated by the fact that ion-rich permafrost on top of drilling sumps and in adjacent lease areas is thawing, as active layer depth is increasing due to enhanced shrub growth [10], liberating base cations, and contributing to elevated ionic concentrations in the nearby water bodies.

**Figure 3:**
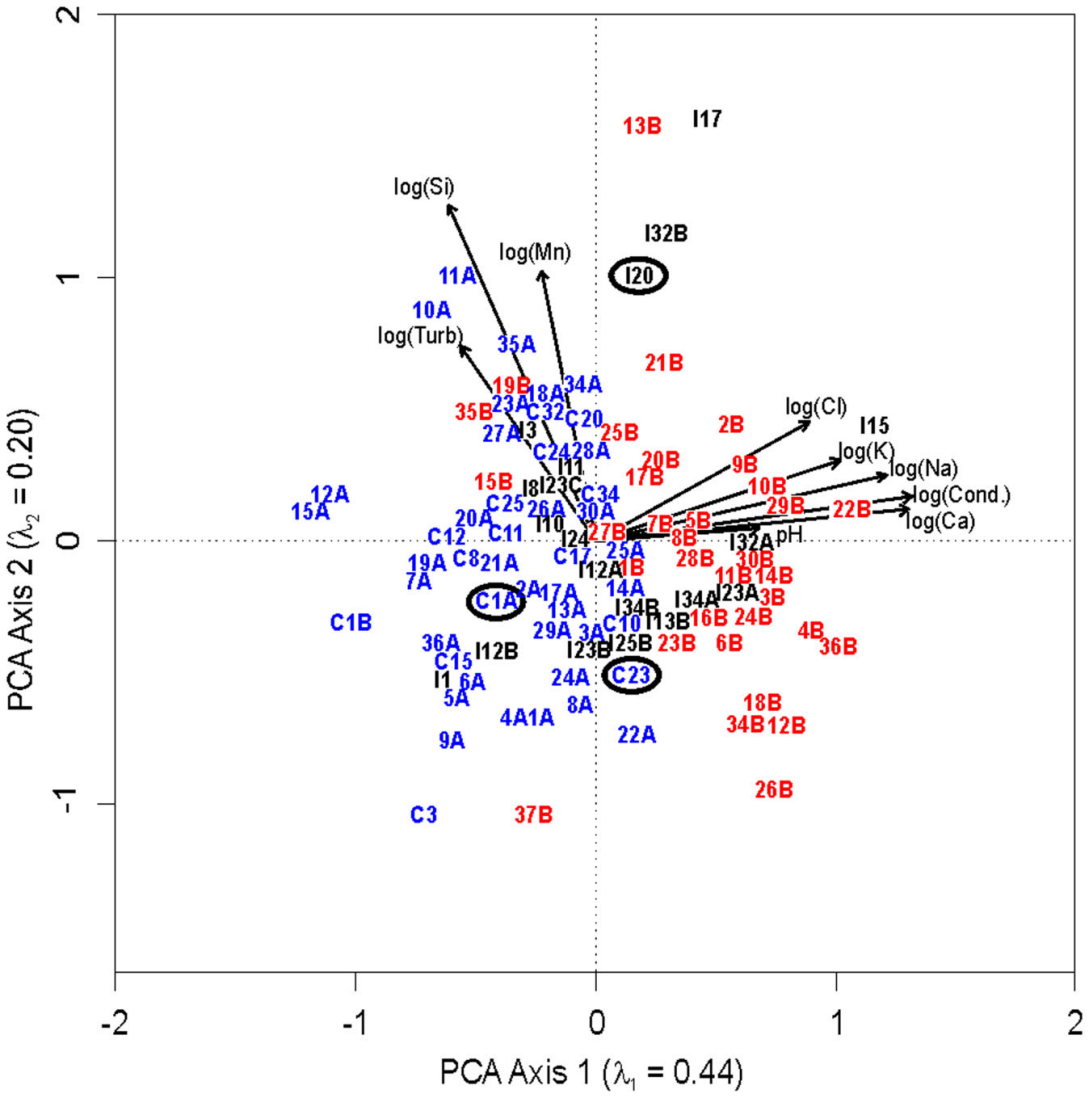
Principal components analysis (PCA) ordination of select chemical variables in the 101 lake dataset. Black labels represent the position of the drilling-sump lakes in the ordination space (n = 20). Red labels represent lakes impacted by retrogressive thaw slumping (n = 34). Blue labels represent control lake ordination results (n = 47). Variables were all normalized using log transformation. Arrows represent importance of given chemical variables in structuring the distribution of sites. Ordination results of study lakes I20, C23, and C1A, from which sediment cores were analyzed, are circled. Si  =  reactive silica.

Principal components analysis (PCA) was used to characterize the water chemistry variation in the 101 study lakes, taking into account the combined importance of all measured environmental variables. The first PCA axis primarily represents a response to ionic strength (Fig. 3), which is not surprising given the importance of these variables in accounting for the differences among groups. Lakes impacted by thaw slumps separate in the PCA plot from control sites primarily based on these variables [16], while the drilling-sump lakes are more widely distributed within the ordination space (Fig. 3). Some drilling-sump lakes, such as I15, I32B, I23A and I17, exhibit environmental conditions similar to sites impacted by large, active permafrost thaw slumps (Fig. 3). Drilling-sump impacted Lake I15 is chemically most similar to thaw-slump affected Lake 10B, which has an active thaw slump impacting over 25% of the lake's catchment [15], despite the lack of any natural geomorphic disturbance in Lake I15's catchment. Other drilling-sump impacted lakes, such as I1 or I12B, are chemically more similar to the control lakes, and thus the sumps in their catchments are likely providing better containment of drilling fluids (Fig. 3). Concentrations of major ions and conductivity appear to be a useful tracer for identifying the influx of materials from deteriorating drilling sumps into nearby lake ecosystems. The variability in the response of drilling-sump impacted lakes is expected, given the range of sump conditions currently observed in the region, with some sumps experiencing significant deterioration while other sites exhibit reasonable to good cover integrity [9]. Exploratory hydrocarbon drilling appears to have resulted in impacts to the freshwater ecosystems of the Mackenzie Delta uplands, in some cases more severe than large-scale, natural, localized geomorphic disturbances, such as retrogressive thaw slump development, which represent a major stressor to these ecosystems.

Due to the lack of long-term biological monitoring data, paleolimnological approaches were used to assess if sump leakage has resulted in any biological changes in sentinel indicator species [31]. Multiple sedimentary proxies were analyzed in radiometrically dated sediment cores from three lakes in the Mackenzie Delta uplands: two sites near Parsons Lake, a location that has undergone significant exploratory hydrocarbon drilling over the last 50 years [6]; and one lake to the south (Fig. 1). Lake I20, impacted by a drilling sump in its catchment, shows elevated ionic water concentrations compared to the majority of the control lakes (Fig. 3), suggesting that drilling fluids may be impacting this lake. PCA (Fig. 3) revealed that some control sites, such as Lake C23, exhibited ion concentrations similar to those of some drilling sump-impacted lakes. Because these lakes have no known history of localized disturbance, we hypothesized that the elevated conductivity levels in C23 are natural, and sedimentary indicators in lake sediments should exhibit no change related to sump development. Lake C1A has conductivity levels more typical of other control lakes (Fig. 3). The analysis of I20 and two control lakes that differ in present-day conductivity will allow any changes in the drilling sump-impacted site to be placed in the context of natural chemical variability in the region.

Concentrations of polycyclic aromatic hydrocarbons did not increase following the construction of the drilling sump near Lake I20 (Text S1, Fig. S3). This suggests that, unlike the impact of large-scale extraction operations such as the Alberta oilsands [4], [5], impacts from exploratory drilling activities in this region of the Arctic do not appear related to contamination by hydrocarbons themselves. This is not surprising, given that most drilling in the delta region utilizes brine-based drilling muds (compared to oil-based), and thus sump contents proportionately contain very little hydrocarbons. Instead, any environmental impacts of these activities are likely to be related to chemicals present in the drilling fluids, notably salts.

Many species of Cladocera (Crustacea, Branchiopda) are considered poor osmoregulators, and thus we hypothesized that changes in ionic strength following drilling-sump failure would result in a shift in the species assemblage. In addition there is evidence that potassium chloride in the concentrations found in drilling fluids produces both lethal and sub-lethal effects on Cladocera in laboratory experiments [13]. Cladocera are a key component of aquatic foodwebs, and thus understanding their response to this environmental stressor is essential.

**Figure 4:**
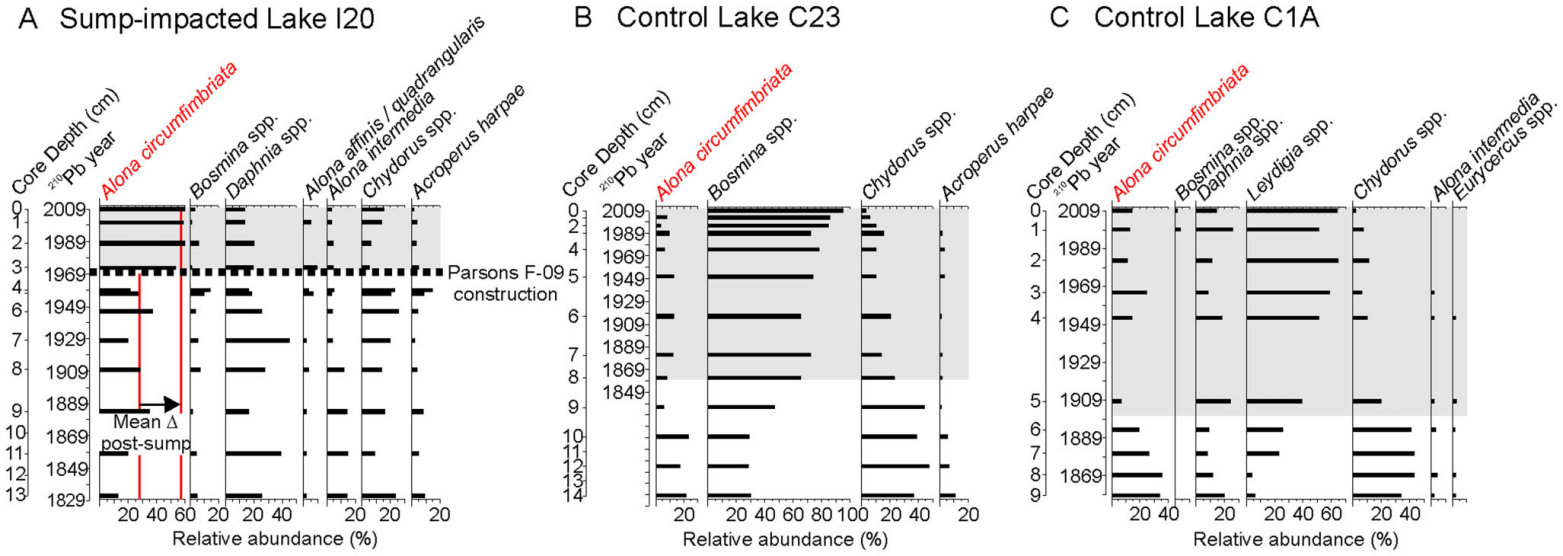
Stratigraphic profile of the most common cladoceran taxa from the three study lakes. Relative abundance diagrams from: lakes A) I20, impacted by drilling sump degradation; and control lakes B) C23; and C) C1A. Species assemblages (x axes) are scaled by relative abundance. Down-core sedimentary profiles (y axes) are scaled by date, based on ^210^Pb radiometric dating techniques, with the depth in the sediment core included as a secondary axis. For all three lakes, two biostratigraphic zones were identified (constrained incremental sum of squares cluster analysis with the broken stick model) and are plotted with the background colour of one zone in grey the other white. The known timing of construction of the compromised drilling sump near Lake I20 (industry ID: Parsons F-09) is included as a horizontal line. The vertical red lines represent the pre- and post-sump construction mean of the relative abundance of the cladoceran *Alona circumfimbriata*.

**Figure 5:**
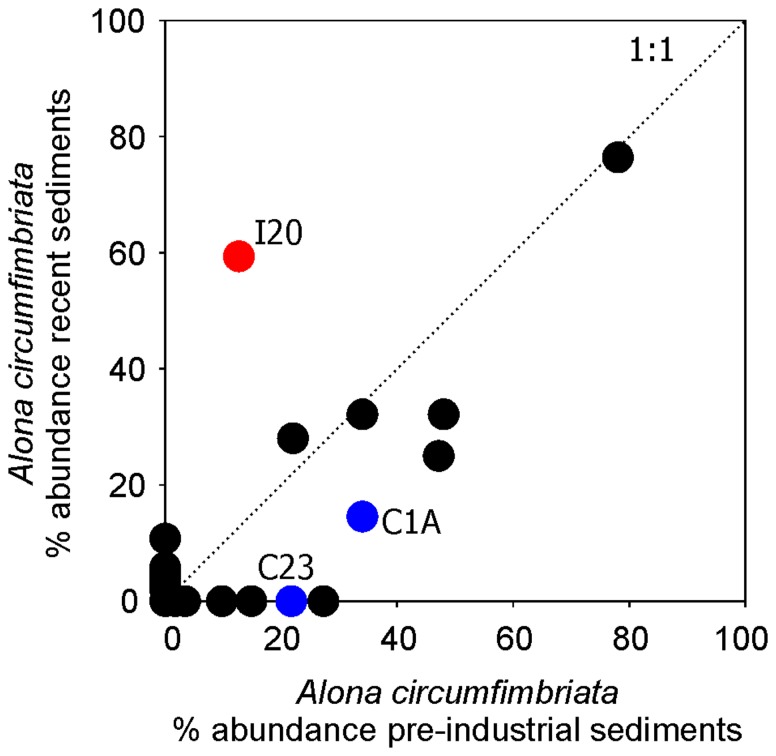
Change in the relative abundance of *Alona circumfimbriata* between present-day and pre-industrial sediment samples. Based on a comparison of the top of the sediment core (representing present-day conditions) with a bottom sample (pre-industrial) from 50 lakes in the Canadian sub-Arctic. Black circles represent 47 lakes from the only regional cladoceran survey in the Canadian sub-Arctic, spanning present-day treeline in the Northwest Territories (data recalculated from [34]). Blue circles represent the control lakes (C23, C1A) from this study. The red circle represents drilling sump-impacted Lake I20. The 1:1 line is also shown.

In Lake I20, an abrupt increase in the relative abundance of *Alona circumfimbriata* subfossils, a taxon which is known to be relatively saline tolerant [32], [33], occurred near the time of sump development in ∼1972 (Fig. 4). In a survey of Cladocera in sub-Arctic lakes spanning treeline in the NWT, this taxon was most common in the highest conductivity sites [34]. While the overall magnitude of this assemblage change is relatively subtle, the timing and rapid nature of this change occurring at the time of (or very soon after) the construction of the sump suggests sump disturbance for at least this site may be primarily related to construction and abandonment practices. No comparable changes in the cladoceran assemblage of either control lake were observed coincident with, or subsequent to, the construction of the sumps near to (but not in the catchment of) those sites, despite the natural variability in ion-related water chemistry (Fig. 3). Instead, the primary changes that do occur in the control lakes are gradual and more indicative of regional warming, which has been extensive in this region and has been inferred to be impacting the cladoceran assemblages in another lake in the Mackenzie Delta [35]. The increase in *A. circumfimbriata* in I20 is unique when analyzed in the context of the only other regional dataset available for Cladocera in the Canadian sub-Arctic [34] (Fig. 5). Sedimentary diatom assemblages recorded no changes inferred to be as a result of drilling-sump containment loss, and instead are responding to climate warming which has been significant in this region (Text S1, Fig. S4). The lack of diatom response to these still relatively subtle changes in lakewater chemistry is not surprising. In lakes impacted by large permafrost thaw slumps, diatom assemblage shifts were inferred to be in response to modifications in aquatic habitat, and not chemical changes [18].

As the sub-Arctic and Arctic continue to warm rapidly, and permafrost temperatures increase, the likelihood of waste containment issues in drilling mud sumps will increase, potentially increasing impacts on aquatic ecosystems. Possible climate-induced increases in evaporation, as have been observed in other high-latitude regions [36], [37], could also lead to reductions in lakewater level, and further concentration of salts. This will be exacerbated by increased ionic flux due to warming and the thaw of containing permafrost. It is therefore possible that the impacts inferred from the comparison of present-day chemistry and long-term sediment records of these lakes in the Mackenzie Delta represent as yet a small component of the potential impact of drilling sumps on lake ecosystems. This is significant due to the fact that, to date, at some sites, the impacts on contemporary limnology have been as large as those associated with spectacular and conspicuous permafrost degradation. Drilling sumps represent only one example of the multiple stressors impacting Arctic lakes, which when combined with other natural and anthropogenic stressors may result in cumulative impacts to aquatic systems. If the contents of drilling sumps leach into the aquatic ecosystem due to loss of containment following permafrost thaw, the quantity and type of materials leaching into nearby lakes may increase and could result in severe, deleterious impacts on aquatic life. As the scale of hydrocarbon exploration and development in the Arctic increases in the coming decades, the potential for widespread contamination of freshwater lakes could result in cascading effects on both aquatic and terrestrial ecosystems, as well as the local indigenous communities which rely on them. In conjunction with work on permafrost conditions at drilling mud sumps [11] our data demonstrate that permafrost has not performed well as a waste containment medium, and thus if total containment of drilling wastes is a primary disposal objective, then alternative methods for waste disposal must be explored.

## Supporting Information

Figure S1 Radioisotopic activity and CRS-model derived age for select intervals from the three analyzed sediment cores.
(TIF)

Figure S2 Boxplots of select environmental variables exhibiting significant differences between *a priori* defined groups. SU – drilling sump lakes; CO – control lakes; SL – thaw slump lakes. In each plot, letters indicate significantly different groups (calculated using a Tukey HSD post-hoc test, following ANOVA run on normalized environmental data).
(TIF)

Figure S3 Profiles of PAH concentrations and inferred primary production in sediment cores from three study lakes. Visual reflectance spectroscopically-inferred chlorophyll *a* (VRS chl *a*: circles) and summed 12 priority polycyclic aromatic hydrocarbon (PAH) concentrations (squares) for study lakes A) I20, B) C23 and C) C1A. The limit of detection for VRS-chl *a*, which includes its diagenetic products, is 0.01 mg/g dry weight, and thus the entire C1A profile is below detection.
(TIF)

Figure S4 Stratigraphic profile of the most common diatom taxa from three study lakes. Relative abundance diagrams from lakes A) I20, impacted by drilling sump failure; and control lakes B) C23 and C) C1A. Down-core sedimentary profiles (y axes) are scaled by date, based on ^210^Pb radiometric dating techniques, with the depth in the sediment core included as a secondary axis. For lakes I20 and C23, two biostratigraphic zones were identified (constrained incremental sum of squares cluster analysis with the broken stick model) and are plotted with the background colour of one zone in grey the other white. For Lake C1A, no significant biostratigraphic zones were identified. The known timing of construction of the leaching drilling sump near Lake I20 (industry ID: Parsons F-09) is included as a horizontal line.
(TIF)

Table S1 Summary statistics of water chemistry data. Sites were sampled in the summers of 2005 and 2007, with samples taken at approximately one metre water depth.
(DOC)

Table S2 Results of similarity percentages (SIMPER) analysis.
(DOC)

Text S1 Supplementary methods, hydrocarbon, diatom and inferred primary production results and discussion.
(DOCX)
