# Supplementary figures and images for: Exploratory Hydrocarbon Drilling Impacts to Arctic Lake Ecosystems
Source: PLoS One. 2013 Nov 6;8(11):e78875. doi: 10.1371/journal.pone.0078875 (PMC3819393; doi:10.1371/journal.pone.0078875)

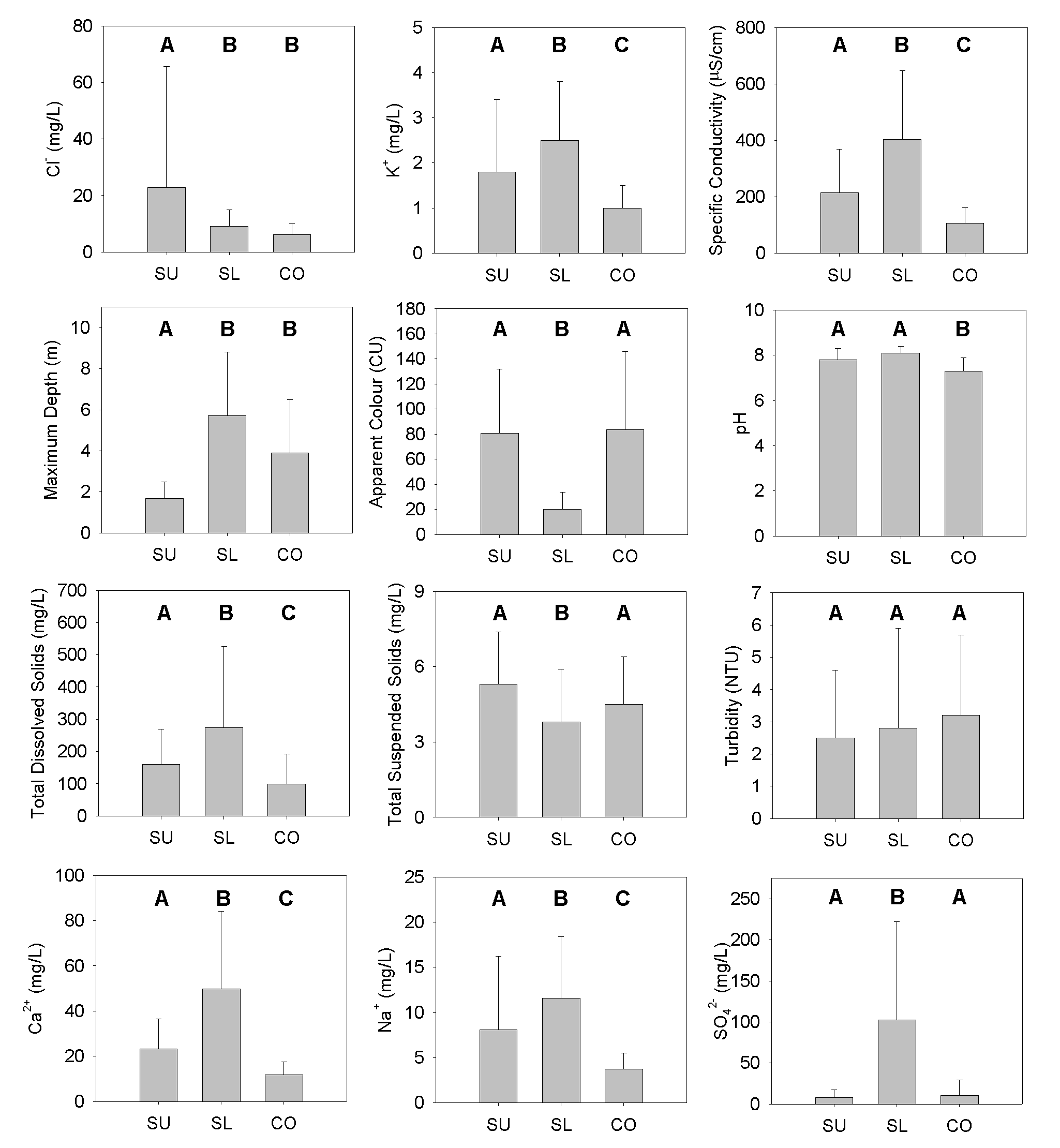

Supplement: Figure S2 — Boxplots of select environmental variables exhibiting significant differences between a priori defined groups. SU – drilling sump lakes; CO – control lakes; SL – thaw slump lakes. In each plot, letters indicate significantly different groups (calculated using a Tukey HSD post-hoc test, following ANOVA run on normalized environmental data). (TIF) [file pone.0078875.s002.tif]

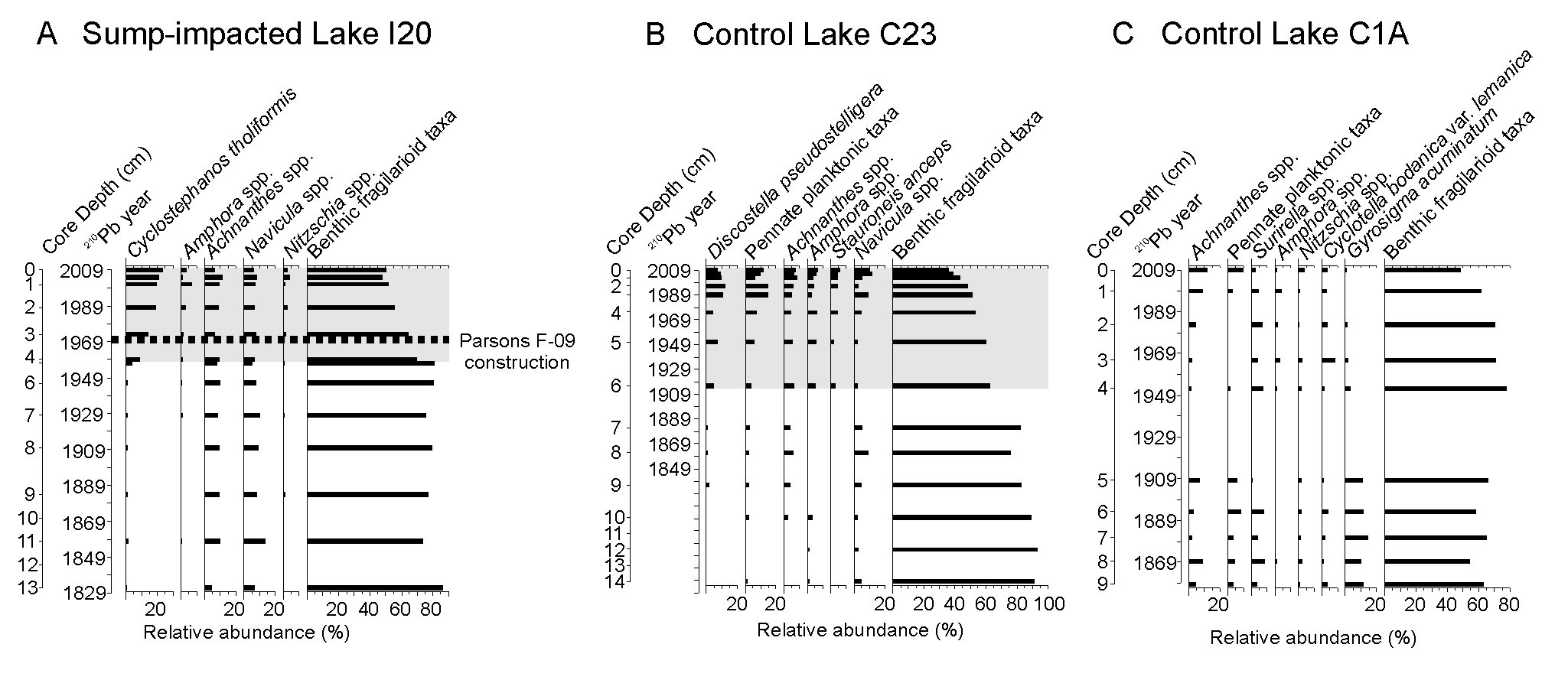

Supplement: Figure S4 — Stratigraphic profile of the most common diatom taxa from three study lakes. Relative abundance diagrams from lakes A) I20, impacted by drilling sump failure; and control lakes B) C23 and C) C1A. Down-core sedimentary profiles (y axes) are scaled by date, based on 210Pb radiometric dating techniques, with the depth in the sediment core included as a secondary axis. For lakes I20 and C23, two biostratigraphic zones were identified (constrained incremental sum of squares cluster analysis with the broken stick model) and are plotted with the background colour of one zone in grey the other white. For Lake C1A, no significant biostratigraphic zones were identified. The known timing of construction of the leaching drilling sump near Lake I20 (industry ID: Parsons F-09) is included as a horizontal line. (TIF) [file pone.0078875.s004.tif]
