# Supplementary material for: Exploratory Hydrocarbon Drilling Impacts to Arctic Lake Ecosystems
Source: PLoS One. 2013 Nov 6;8(11):e78875. doi: 10.1371/journal.pone.0078875 (PMC3819393; doi:10.1371/journal.pone.0078875)
Supplement: Table S1 — Summary statistics of water chemistry data. Sites were sampled in the summers of 2005 and 2007, with samples taken at approximately one metre water depth. (DOC) [file pone.0078875.s005.doc]

**Table S1: Summary statistics of water chemistry data. Sites were sampled in the summers of 2005 and 2007, with samples taken at approximately one metre water depth.**

|  | Sur Area*  (ha) | Catch Area*  (ha) | Z_max_*  (m) | Alk*  (mg/l) | Col*  (CU) | Cond*  (µS/cm) | pH | TDS*  (mg/l) | Turb*  (NTU) | Ca  (mg/l) | Mg  (mg/l) | Na  (mg/l) | K  (mg/l) | Cl  (mg/l) | SO_4_  (mg/l) | Fe  (µg/l) | Mn  (µg/l) |
| --- | --- | --- | --- | --- | --- | --- | --- | --- | --- | --- | --- | --- | --- | --- | --- | --- | --- |
| Drilling-sump lakes (n = 20) | | | | | | | | | | | | | | | | | |
| Mean | 15.8 | 83.5 | 1.7 | 58 | 81 | 214 | 7.8 | 160 | 2.5 | 23.2 | 10 | 8.1 | 1.8 | 22.8 | 8 | 477 | 46 |
| Median | 4.8 | 27.3 | 1.5 | 49 | 68 | 144 | 7.8 | 132 | 1.8 | 18.9 | 7 | 4.9 | 1.3 | 8.0 | 6 | 394 | 31 |
| STD | 22.4 | 162.9 | 0.8 | 35 | 51 | 155 | 0.5 | 110 | 2.1 | 13.2 | 6 | 8.1 | 1.6 | 42.9 | 9 | 397 | 50 |
| Max | 85.7 | 749.4 | 3.4 | 145 | 243 | 738 | 9.0 | 554 | 9.6 | 54.8 | 27 | 37.3 | 6.7 | 198.0 | 40 | 1570 | 214 |
| Min | 0.5 | 15.5 | 1.0 | 12 | 19 | 58 | 6.9 | 61 | 0.5 | 6.9 | 33 | 1.8 | 0.5 | 1.3 | 1 | 50 | 9 |
| Thaw slump-affected lakes (n = 34) | | | | | | | | | | | | | | | | | |
| Mean | 13.1 | 50.6 | 5.7 | 90 | 20 | 404 | 8.1 | 275 | 2.8 | 49.9 | 17 | 11.6 | 2.5 | 9.1 | 102 | 186 | 36 |
| Median | 6.2 | 26.0 | 5.0 | 86 | 16 | 354 | 8.1 | 192 | 1.5 | 42.3 | 13 | 10.9 | 2.1 | 6.7 | 52 | 131 | 21 |
| STD | 22.6 | 62.6 | 3.1 | 41 | 14 | 243 | 0.3 | 251 | 3.1 | 34.2 | 12 | 6.8 | 1.3 | 5.9 | 120 | 190 | 47 |
| Max | 116.5 | 254.4 | 14.2 | 174 | 50 | 1000 | 8.4 | 1330 | 15.8 | 146.0 | 53 | 30.8 | 6.1 | 20.6 | 406 | 855 | 253 |
| Min | 1.2 | 4.7 | 1.7 | 20 | 5 | 84 | 7.2 | 52 | 0.5 | 6.9 | 3 | 2.3 | 0.4 | 1.9 | 2 | 50 | 4 |
| Control lakes (n = 47) | | | | | | | | | | | | | | | | | |
| Mean | 5.0 | 64.8 | 3.9 | 29 | 84 | 107 | 7.3 | 99 | 3.2 | 11.8 | 4 | 3.7 | 1.0 | 6.1 | 10 | 895 | 42 |
| Median | 2.9 | 23.0 | 3.3 | 26 | 74 | 102 | 7.5 | 84 | 2.5 | 11.3 | 4 | 3.3 | 1.0 | 5.9 | 4 | 505 | 25 |
| STD | 6.3 | 151.9 | 2.8 | 18 | 62 | 54 | 0.6 | 93 | 2.5 | 5.8 | 2 | 1.8 | 0.5 | 3.9 | 19. | 1058 | 71 |
| Max | 32.5 | 749.4 | 11.2 | 94 | 266 | 287 | 8.2 | 694 | 12.6 | 27.9 | 9 | 7.7 | 2.5 | 13.6 | 118 | 5960 | 482 |
| Min | 0.5 | 3.9 | 1.0 | 4 | 5 | 29 | 4.3 | 40 | 0.3 | 4.2 | 1 | 0.8 | 0.2 | 0.9 | 1 | 0 | 2 |
