# Supplementary material for: Exploratory Hydrocarbon Drilling Impacts to Arctic Lake Ecosystems
Source: PLoS One. 2013 Nov 6;8(11):e78875. doi: 10.1371/journal.pone.0078875 (PMC3819393; doi:10.1371/journal.pone.0078875)
Supplement: Table S2 — Results of similarity percentages (SIMPER) analysis. (DOC) [file pone.0078875.s006.doc]

Table S2: Results of similarity percentages (SIMPER) analysis.

| Variable | Dissimilarity Contribution (%) | Cumulative Contribution (%) |
| --- | --- | --- |
| Drilling sump and Control |  |  |
| Chloride | 18.54 | 18.54 |
| Silica | 15.60 | 34.13 |
| Maximum depth | 11.14 | 45.28 |
| Manganese | 10.76 | 56.04 |
| Sodium | 8.56 | 64.59 |
| Drilling sump and Permafrost thaw |  |  |
| Silica | 18.11 | 18.11 |
| Chloride | 13.25 | 31.36 |
| Manganese | 12.43 | 43.79 |
| Maximum depth | 12.25 | 56.04 |
| Calcium | 8.99 | 65.03 |
| Permafrost thaw and Control |  |  |
| Calcium | 15.42 | 15.42 |
| Silica | 14.31 | 29.73 |
| Specific conductivity | 13.92 | 43.65 |
| Sodium | 11.36 | 55.01 |
| Manganese | 10.02 | 65.03 |
