# Supplementary material for: Exploratory Hydrocarbon Drilling Impacts to Arctic Lake Ecosystems
Source: PLoS One. 2013 Nov 6;8(11):e78875. doi: 10.1371/journal.pone.0078875 (PMC3819393; doi:10.1371/journal.pone.0078875)
Supplement: Text S1 — Supplementary methods, hydrocarbon, diatom and inferred primary production results and discussion. (DOCX) [file pone.0078875.s007.docx]

Text S1. Supplementary methods, hydrocarbon, diatom and inferred primary production results and discussion.

Supplementary Methods

Analyses of water chemistry:

Water samples were collected from the central portion of each lake approximately 1 m below the surface, and were kept cool until transferred to the Taiga Environmental Laboratory (Aboriginal Affairs and Northern Development Canada) in Yellowknife, NT. The Taiga laboratory is an ISO/IEC 17025 accredited laboratory and a member of the Canadian Association for Laboratory Accreditation (CALA). Accreditation results can be viewed by entering member number 2635 on the CALA website. The Taiga Environmental Laboratory also regularly participates in QA/QC activities and exchanges with other accredited laboratories, including the National Laboratory for Environmental Testing (NLET; Environment Canada; Burlington, ON). Chemical determination followed a variety of standard protocols depending on specific analyte. Major ions were measured using ion chromatography following modified method SM4110B. Metals were analyzed by inductively coupled plasma – mass spectrometry following EPA method 200.8. Turbidity was analyzed by nephelometry following modified method SM2130B. True and apparent colour were analyzed via spectrophotometer following method SM2120C. Only chemical variables where the majority of samples occurred above the detection limit were included in further analyses.

Sediment collection, radiometric dating and sediment age determination:

Sediment cores from the three study lakes were collected from a raft in the central, deepest portion of the lake using a Glew-type gravity corer (internal diameter: 7.62 cm; [1]) and sectioned at 0.5 cm resolution by vertical extrusion [2]. Sediment core intervals were dated using ^210^Pb and ^137^Cs radiometric techniques, via gamma spectroscopy [3, 4]. Selected sediment intervals were lyophilized, homogenized, entombed in epoxy resin and allowed to equilibrate for a minimum of two weeks. Samples were then analyzed for ^210^Pb, ^214^Bi and ^137^Cs activity using an Ortec^®^ well-type Ge crystal detector for 80,000 seconds each. Sediment age determination was conducted using the constant rate of supply (CRS) model [5], with the supported ^210^Pb activity determined via the method established by [6]. The radioisotopic activities, as well as respective CRS-derived sediment ages, are presented in Fig. S1. For each core the relationship between the cumulative dry mass of the sediment interval and the unsupported ^210^Pb activity (log transformed) was examined and found to be strong (r > 0.8).

Supplementary PAH methods:

Wet sediments were homogenized and mixed in an approximate 1:1 ratio with Agilent brand Hydromatrix^©^. The samples were spiked with ^13^C labelled PAHs (Cambridge Isotope Laboratories). PAHs were extracted from the wet sediments using accelerated solvent extraction (ASE, Dionex) at 100˚C with hexane:dichloromethane (DCM), followed by 35% acetone:65% hexane. The non-polar extract (PAHs, hexane and DCM) was separated from the polar extract (water and acetone) by combining the entire sample with DCM, 3% NaCl and saturated Na_2_SO_4_ in a series of liquid-liquid extractions. Sulphur compounds and pigments were removed from the final extract on an Agilent 1100 Preparative Liquid Chromatograph using Waters Envirogel Columns and USEPA method 3640A. The samples were evaporated to 1ml and fractioned, according to USEPA method 3630C, on Davisil 635 silica (60-100 mesh, pore size 60). The PAH fraction was analyzed by gas chromatography (Agilent 6890) and mass spectrometry (Agilent 5973). 1µl injections were made in pulsed splitless mode at 280˚C on a DB-XLB 30m x 0.18µm x 180µm column. An initial oven temperature of 60˚C was held for 2 minutes then increased at a rate of 6˚C per minute to 300˚C and held for 10 minutes. A constant flow rate of 39cm per second of helium was used for a total run time of 52 minutes. The mass spectrometer was set to have a transfer line temperature of 280˚C, with a source temperature of 230˚C and quadrapole temperature of 150˚C.

The following fragments were monitored for PAHs in Single Ion Monitoring (SIM) mode: m/z 128 for naphthalene, m/z 152 for acenapthylene, m/z 153 for acenapthene, m/z 166 for fluorine, m/z 178 for phenanthrene and anthracene, m/z 202 for fluoranthene and pyrene, m/z 228 for benz(a)anthracene and chrysene, m/z 240 for chrysene, m/z 252 for benzo(b)fluoranthene, benzo(k)fluoranthene and benzo(a)pyrene, m/z 278 for indeno(123-cd)pyrene and deibenz(a,h)anthracene, and m/z 276 for benzo(g,h,i)perylene. Indeno(123-cd)pyrene and dibenz(a,h)anthracene were combined because of poor peak resolution. Quantification was performed using ^13^C labelled PAHs (Cambridge Isotope Laboratories) for all of the compounds listed above.

The following fragments were monitored for alkylated PAHs in SIM mode: m/z 142 for 1- and 2-methylnapthalene, m/z 156 for C2-napthalene, m/z 170 for C3-napthalene, m/z184 for C4-napthalene, m/z 180 for C1-fluorine, m/z194 for C2-fluorine, m/z 208 for C3-fluorine, m/z 192 for C1-phenanthrene and C1-anthracene, m/z 206 for C2-phenanthrene and C2-anthracene, m/z 220 for C3-phenanthrene and C3-anthracene, m/a 234 for C4-phenanthrene and C4-anthracene, m/z 216 for C1-fluoranthene and C1-pyrene, m/z 230 for C2-fluoranthene and C2-pyrene, m/z for 244 for C3-fluroanthene and C3-pyrene, m/z 258 for C4-fluoranthene and C4-pyrene, m/z 242 for C1-benz(a)anthracene and C1-chrysene, m/z 256 for C2-benz(a)anthracene and C2-chrysene, m/z C3-benz(a)anthracene and C3-chrysene, m/z 266 for C1-benzfluoranthene and C1-benzopyrene , m/z 280 for C2-benzofluorantene and C2-benzopyrene. Quantification was performed using ^13^C labelled non-alkylated PAHs (Cambridge Isotope Laboratories) for all of the compounds listed above.

Supplementary Results and Discussion

Present-day lakewater chemical comparisons:

Comparisons of present-day physical and chemical limnological variables reveal major differences between the three *a priori* defined groups (all comparisons made using two-way ANOVA, df=2, 98, followed by Tukey HSD post-hoc test run on normalized data) (Fig. S2). Drilling sump lakes exhibited significantly higher Cl^-^ concentrations than either permafrost thaw or control lakes (F=7.91, p<0.001). Permafrost thaw lakes exhibited significantly increased K^+^ (F=23.6, p<0.001), Ca^2+^ (F=50.2, p<0.001), and Na^+^ (F=30.7, p<0.001) concentrations, specific conductivity (F=47.3, p<0.001) and total dissolved solids (F=18.8, p<0.001) than the drilling sump lakes which were all significantly greater than in the control lakes (Fig. S2). The drilling sump lakes were significantly shallower than the permafrost thaw or control lakes (F=19.7, p<0.001). Lakes with permafrost thaw slumps had significantly higher apparent colour (F=37.0, p<0.001), SO_4_^2-^ concentration (F=36.0, p<0.001) and lower total suspended solids (F=4.52, p=0.01) than the other two groups. The control lakes had lower pH than either the drilling sump or permafrost thaw lakes (F=22.1, p<0.001). No significant difference was observed in water turbidity between the three groups (F=1.46, p=0.24). Analysis of similarity (ANOSIM) and similarity percentages (SIMPER) were used in order to assess differences in the *a priori* defined groups, as well as determine which variables contributed to any statistical differences. SIMPER analysis, run on a Euclidean distance matrix, determined that Cl^-^, Si, and Mn concentrations, as well as maximum depth, contributed significantly to the difference between the drilling sump and control lakes as well as the drilling sump and permafrost thaw lakes (Table S2). Ca^2+^, Si, and Mn concentrations, as well as specific conductivity, were found to contribute to the dissimilarity between the permafrost thaw and control lakes, though notably chloride concentration was not observed to be important (Table S2).

Polycyclic aromatic hydrocarbons (PAHs):

Sedimentary concentrations of the 12 priority PAHs, including the most common PAHs from petrogenic sources, did not change at the time of, or subsequent to, drilling sump construction in any of the three study lakes (Fig. S3). Included in this summation are the following compounds: phenanthrene, anthracene, fluoranthene, pyrene, benz[a]anthracene, chrysene, benzo[b]fluoranthene, benzo[k]fluoranthene, benzo[a]pyrene, indeno[1,2,3-cd]pyrene, dibenz[a,h]anthracene, and benzo[g,h,i]perylene. This suggests that, unlike the impact of large-scale extraction operations such as the Alberta oilsands [7, 8], impacts from exploratory drilling activities in this region of the Arctic do not appear related to contamination by hydrocarbons themselves. This is not surprising, given that these exploratory operations extract comparatively few hydrocarbons during the process of test well development, and given that relatively high, stable background PAH concentrations were observed in the sediments of all three lakes throughout the past few hundred years (Fig. S3).

Sedimentary diatoms:

Sedimentary diatom assemblages from Lake I20, impacted by a failing drilling sump, do not show any response coincident with, or subsequent to, sump construction (Fig. S4). The modern assemblage is dominated by the planktonic taxon *Cyclostephanos tholiformis*, as well as a variety of small benthic taxa of the group *Fragilaria sensu lato* (primarily *Staurosirella pinnata*, *Staurosira venter* and *Pseudostaurosira brevistriata*). Prior to approximately 1950, when a gradual increase in *C. tholiformis* began, the assemblage was dominated by small benthic fragilarioids at greater than 70% relative abundance (Fig. S4). Similarly, in Lake C23, the assemblage is dominated by small benthic fragilarioid taxa throughout the recent past. At ~1910 an increase in planktonic taxa, including *Discostella pseudostelligera* (synonymous with *Cyclotella pseudostelligera*), as well as a number of pennate planktonic taxa occurred, coincident with the onset of warming in this region as recorded by several independent proxies [9]. The primary diatom changes in both I20 and control Lake C23 occur earlier than the known construction of drilling sumps in the vicinity of the lakes, and are characterized by increased planktonic diatom abundance, indicative of climate warming [10], similar to the timing and nature observed in many other lakes in this region [9]. The changes in diatom assemblages in I20 and C23 occur simultaneously with increases in inferred primary production (Fig. S3), suggesting an influence of warming on overall primary production in these lakes. No major changes were observed in the diatom record from C1A (Fig. S4), which is composed of an assemblage dominated by small *Fragilaria sensu lato* taxa as well as a variety of other periphytic and benthic species. The inferences of primary production in C1A were below detection limits (Fig. S3). In no lake was an increase in saline-tolerant taxa observed, such as those recorded following saline intrusion in the nearby outer Mackenzie Delta [11]. The lack of diatom response to changes in lakewater chemistry as a result of drilling sump failure is not surprising as diatoms were found to respond to changes in aquatic habitat, and not chemistry, following the development of large permafrost thaw slumps; systems which our results show have similar chemical compositions.

References

1. Glew JR (1989) A new trigger mechanism for sediment samplers. J Paleolimnol 2:241-243.

2. Glew JR (1988) A portable extruding device for close interval sectioning of unconsolidated core samples. J Paleolimnol 1:235-239.

3. Appleby PG (2001) Chronostratigraphic techniques in recent sediments. In: Last WM, Smol JP, editors. Tracking Environmental Changes Using Lake Sediments. Volume 1: Basin Analysis, Coring, and Chronological Techniques. Dordrecht: Kluwer Academic Publishers. pp. 171-203.

4. Schelske CL, Peplow A, Brenner M, Spencer CN (1994) Low-background gamma counting: applications for ^210^Pb dating of sediments. J Paleolimnol 10:115-128.

5. Appleby PG, Oldfield F (1978) The calculation of ^210^Pb dates assuming a constant rate of supply of unsupported ^210^Pb to the sediments. Catena **5:**1-8.

6. Binford MW (1990) Calculation and uncertainty analysis of ^210^Pb dates for PIRLA project lake sediment cores. J Paleolimnol 3:253–267.

7. Kelly EN, Short JW, Schindler DW, Hodson PV, Ma M, et al. (2009) Oil sands development contributes polycyclic aromatic compounds to the Athabasca River and its tributaries. Proc Natl Acad Sci USA 106:22346–22351.

8. Kurek J, Kirk JL, Muir DCG, Wang X, Evans MS, et al. (2013) Legacy of a half century of Athabasca oil sands development recorded by lake ecosystems. Proc Natl Acad Sci USA 110:1761-1766.

9. Thienpont JR, Rühland KM, Pisaric MFJ, Kokelj SV, Kimpe LE, et al. (2013) Biological responses to permafrost thaw slumping in Canadian Arctic lakes. Freshw Biol 58:337-353.

10. Rühland KM, Paterson AM, Smol JP (2008) Hemispheric-scale patterns of climate-induced shifts in planktonic diatoms from North American and European lakes. Global Change Biol 14:2740-2745.

11. Thienpont JR, Johnson D, Nesbitt H, Kokelj SV, Pisaric MFJ, Smol JP (2012) Arctic coastal freshwater ecosystem responses to a major saltwater intrusion: a landscape-scale palaeolimnological analysis. The Holocene 22:1447-1456.
